# Coronary artery properties in atherosclerosis: A deep learning predictive model

**DOI:** 10.3389/fphys.2023.1162436

**Published:** 2023-04-05

**Authors:** Ricardo Caballero, Miguel Ángel Martínez, Estefanía Peña

**Affiliations:** ^1^ Aragón Institute of Engineering Research (I3A), University of Zaragoza, Zaragoza, Spain; ^2^ Biomedical Research Networking Center in Bioengineering, Biomaterials and Nanomedicina (CIBER-BBN), Madrid, Spain

**Keywords:** cardiovascular diseases, atheroma plaque, *in silico* modeling, deep learning, artificial neural network

## Abstract

In this work an Artificial Neural Network (ANN) was developed to help in the diagnosis of plaque vulnerability by predicting the Young modulus of the core (*E*_*core*_) and the plaque (*E*_*plaque*_) of atherosclerotic coronary arteries. A representative *in silico* database was constructed to train the ANN using Finite Element simulations covering the ranges of mechanical properties present in the bibliography. A statistical analysis to pre-process the data and determine the most influential variables was performed to select the inputs of the ANN. The ANN was based on Multilayer Perceptron architecture and trained using the developed database, resulting in a Mean Squared Error (MSE) in the loss function under 10^–7^, enabling accurate predictions on the test dataset for *E*_*core*_ and *E*_*plaque*_. Finally, the ANN was applied to estimate the mechanical properties of 10,000 realistic plaques, resulting in relative errors lower than 3%.

## 1 Introduction

Cardiovascular diseases (CVDs) are the leading cause of death in the world. An estimated 17.9 million people died from CVDs in 2019, representing 32% of all global deaths. Atherosclerosis is one of the most common CVDs, causing more than 50% of the sudden deaths (Wilkins et al., 2017; Roth et al., 2020; World Health Organization, 2021). This pathology may start at a premature age (Napoli et al., 1997; Napoli et al., 1999; Pederiva et al., 2021), but, over time, it can result in acute events such as strokes and heart attacks. One of the main causes that trigger atherosclerosis is endothelial damage. From a mechanical point of view, a decrease in Wall Shear Stress (WSS) will eventually reshape the endothelial cells into a more circular shape causing an increase in their permeability (Malek et al., 1999; Esper et al., 2006).

**FIGURE 1:**
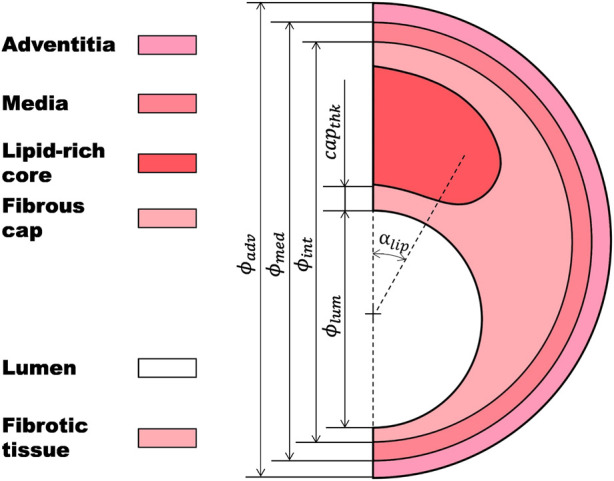
Idealized atherosclerotic coronary artery including lipid-rich core, fibrotic tissue, fibrous cap, tunica media, and tunica adventitia.

An atherosclerotic coronary artery is usually divided into the following parts (Figure 1): a necrotic lipid-rich core or fatty tissue; a fibrotic tissue, which is the result of a thickening of the tunica intima due to a migration of synthetic smooth muscle cells from the tunica media; the fibrous cap, which is the thin layer of fibrotic tissue separating the lipid-rich core from the lumen; the tunica media and the tunica adventitia (Falk et al., 1995; Lee and Libby, 1997; Virmani et al., 2000; Akyildiz et al., 2018). The major risk of a plaque is when there is an evident risk that the fibrous cap ruptures, releasing the necrotic content into the lumen, and causing a thrombus that may result in a myocardial infarction (Finn et al., 2010; Stefanadis et al., 2017).

The mechanical characterization of atherosclerotic arteries has been shown to be valuable for diagnosing vulnerable plaques. Additive manufacturing can leverage this mechanical characterization to create replicas that can be useful in surgical simulation (Henriques et al., 2023). In addition, other approaches, such as the one proposed by Gold et al. (2019), highlight the importance of mechanical characterization of each layer of the artery in developing predictive models of vascular pathophysiology.

In order to make a diagnosis of this pathology, the gold standard technique is intravascular ultrasound echography (IVUS) (Moore et al., 1998; Nair et al., 2002; Nasu et al., 2006; Gogas et al., 2011; Li et al., 2014). Using this procedure in combination with endovascular elastography (EVE), it is possible to estimate artery wall strains and, therefore, predict those areas more prone to rupture (Maurice et al., 2004; Maurice et al., 2005; 2007). In addition, several techniques exist to detect vulnerable plaques (VPs) by predicting their morphology and the type of tissue conforming the plaque, such as the intraluminal ultrasonic palpation imaging technique (Gómez et al., 2019), which combines the radio-frequency technique with Finite Element Analysis (FEA); virtual histology, based on the spectral analysis of retro-dispersed radio-frequency ultrasounds (Moore et al., 1998; Kovarnik et al., 2011; Layland et al., 2011; De Graaf et al., 2013); or near-infrared spectroscopy (NIRS), that quantifies the lipid content in the atheroma plaque (Erlinge et al., 2021). However, an approach performed with adult mini-pigs prone to atherosclerosis showed that necrotic core sizes determined by virtual and real histology were different, thus questioning the capacity of virtual histology to detect prone plaques (Thim, 2010). Moreover, none of these techniques have the capacity to quantify the mechanical properties of the arterial wall, which is essential to estimate stress distribution (Akyildiz et al., 2011; Wang et al., 2019). One promising approach is the iMOD elastography (Le Floc’h et al., 2010), which developed several computational algorithms to rebuild elasticity strain maps inside the wall based on the prediction of the stress maps using IVUS-derived techniques. Since 2009, some remarkable improvements have been developed, such as two powerful stability plaque bio-markers of the coronary artery based on radial strains and its gradient, which allow Young’s modulus to be obtained under linear isotropic elasticity, plane strain, and incompressibility hypothesis (Le Floc’h et al., 2009; Tacheau et al., 2016; Gómez et al., 2019). However, all these methods share a common issue: the waiting time between acquiring the image and obtaining the results from the computational model is prolonged due to the need to optimize the problem specific to the patient.

Recently, a new promising study used a combination of magnetic resonance imaging (MRI), FEA, and a Bayesian optimization process for material property assessment under physiological loading conditions (Torun et al., 2022). This study demonstrated the feasibility of estimating material properties through an optimization algorithm. However, its applicability is limited by the small number of specimens tested and the fact that the specimens were *ex-vivo*.

In Wang et al. (2023), the mechanical properties were predicted *in-vivo* starting from IVUS images, where a close link between plaque morphological characteristics and mechanical properties was reported, but the statistical approach of this work was performed with little data (n = 32).

On the contrary, Deep Learning (DL) frameworks have been trained using large databases to predict the mechanical properties of living tissue. For instance, Ma et al. (2022) used DL to predict the elastic modulus of 3D-printed lattice structures. Another noteworthy study employed a 5-layer fully connected neural network to extract tissue optical properties (Hokr and Bixler, 2021).

Therefore, the aim of this project is to propose an approach that may help in the diagnosis of VPs by estimating the *in-vivo* patient-specific mechanical properties (*E*_*core*_ and *E*_*plaque*_) of the plaque constituents. For this reason, we developed a large *in silico* database and explored the utility of Artificial Neural Networks (ANNs) in elastic properties prediction of the atheroma plaque components. Similar machine learning techniques have been previously used to predict the maximum principal stress as one of the most common mechanical predictors for plaque vulnerability (Cilla et al., 2012). However their approach was based on the geometry, but artery mechanical properties were not considered.

## 2 Methods

**FIGURE 2:**
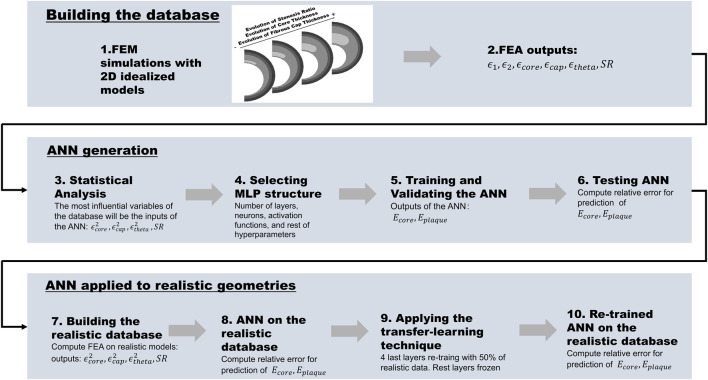
The workflow is divided into building the database, generating the ANN, and applying it to realistic geometries: 1) Building the idealized database and performing the FEA simulations; 2) Selecting the outputs of the FEA (*ϵ*_1_, *ϵ*_2_, *ϵ*_*core*_, *ϵ*_*plaque*_, *ϵ*_*theta*_, and *SR*); 3) Statistical analysis of the FEA output variables and selection of the inputs of the ANN ( $\epsilon_{\mathrm{core}}^{2}$ , $\epsilon_{\mathrm{plaque}}^{2}$ , $\epsilon_{\mathrm{theta}}^{2}$ , and *SR*); 4) Selecting MLP structure; 5) Training and validating the ANN; 6) Testing the ANN by computing the relative error in the prediction of *E*_*core*_ and *E*_*plaque*_; 7) Building the realistic database; 8) Application of the ANN to the realistic database; 9) Application of transfer-learning technique; 10) Application of the transfer-learned ANN to the realistic database and computation of the relative errors.

Figure 2 shows the workflow used to develop a computational framework for the estimation of *E*_*core*_ and *E*_*plaque*_ of a patient-specific atheroma plaque. Based on the information taken from the IVUS images previously published (Le Floc’h et al., 2009) and the anatomy of the atherosclerotic coronary artery, we performed the Finite Element Method (FEM) on 540 different 2D idealized geometries. As seen in other works (Nong et al., 2022; Shen et al., 2022; Le Floc’h et al., 2009; Finet et al., 2004), 2D models are commonly used when artery geometries are obtained from the segmentation of IVUS images. One of the key advantages of using 2D models for studying the mechanical properties of atherosclerotic coronary arteries is that the IVUS images provide 2D cross-sectional information of the coronary artery geometry. Furthermore, 2D models are favored over 3D models as they offer a more streamlined and efficient analysis process, requiring fewer computational resources and yielding quicker results. Subsequently, a statistical analysis was carried out on the outputs of the FEA (strains), in order to select the most influential variables on *E*_*core*_ and *E*_*plaque*_ as the inputs of the ANN. We then trained and tested the ANN by minimizing a loss function (MSE), and computed the relative error in the prediction of the mechanical properties of the training and testing datasets. Finally, we applied the ANN to estimate the mechanical properties of 10,000 plaque geometries and computed the relative error.

### 2.1 Database generation

The idealized 2D geometry of the atherosclerotic coronary artery was divided into four main parts: a lipid-rich core; a thickened tunica intima, which is considered as a fibrotic tissue (where the fibrous cap is the most critical area); the tunica media; and the tunica adventitia (Figure 1). The main geometrical characteristics of the base geometry were: the diameter of the lumen (*ϕ*_*lum*_ = 3.6 *mm*), the diameter of the intima layer (*ϕ*_int_ = 5.596 *mm*), the diameter of the media layer (*ϕ*_*med*_ = 6.05 *mm*), the diameter of the adventitia layer (*ϕ*_*adv*_ = 6.5 *mm*), the thickness of the fibrous cap (*cap*_*thk*_ = 65 *μm*), the angle of the lipid-rich core (*α*_*lipid*_ = 60^*o*^), and the stenosis ratio (*SR* = 70%) (Equation 1).

$$SR=\frac{A_{\mathrm{plaque}}}{A_{\mathrm{plaque}}+A_{\mathrm{lumen}}}\cdot 100$$ (1)

where *A*_*plaque*_ and *A*_*lumen*_ are the area of the plaque and the lumen respectively.

In order to build a solid database, 540 distinct idealized geometries were generated by varying the following parameters (Table 1): nine variations of stenosis ratio (*SR* = 40%–80%) (Finn et al., 2010), ten variations of the thickness of the fibrous cap (*cap*_*thk*_ = 65 *μm* − 300 *μm*) (Loree et al., 1992) and six variations of the thickness of the lipid-rich core (*core*_*thk*_ = 300 *μm* − 800 *μm*) (Virmani et al., 2000).

**TABLE 1:** Proposed ranges for each variable of the model composing the database. Mechanical properties (*E*_*core*_ and *E*_*plaque*_) in kPa. Geometrical variables (*SR*, *cap*_*thk*_ and *core*_*thk*_) in % and *μm* respectively.

Variable	Range	N° param	Step
*E*_*core*_ (*kPa*)	1–100	300	logarithmic variation
*E*_*plaque*_ (*kPa*)	390–1,200	10	fixed (90)
*SR* (%)	40–80	9	fixed (5)
*cap*_*thk*_ (*μm*)	65–300	10	variable (user-selected)
*core*_*thk*_ (*μm*)	300–800	6	fixed (10)

Due to the high variability found in experimental studies on the coronary artery, setting the mechanical properties of each area in the model was a challenging task. Therefore, in this study, we established the range of Young’s modulus for the lipid-rich core (*E*_*core*_ = 1–100 *kPa*) and the fibrotic tissue (*E*_*plaque*_ = 390–1,200 *kPa*) after conducting a thorough analysis of the mechanical properties reported by different authors (Akyildiz et al., 2018; Baldewsing et al., 2004; Cheng et al., 1993; Finet et al., 2004; Le Floc’h et al., 2009; Peña et al., 2021; Gómez et al., 2019). We proposed the ranges of material properties with the aim of covering all possible variability (Table 1). In addition, given the greater variability found in the *E*_*core*_ range, we performed 300 variations with a variable step that followed a logarithmic function, in order to obtain a higher density of data in the lower part of the range. On the other hand, only 10 variations of *E*_*plaque*_ were accomplished with a fixed step of 90 *kPa*, as the variability found in this tissue was lower.

As our objective was to predict the Young’s modulus for both the lipid-rich core and fibrotic tissue, which were assumed to be isotropic, we selected the widely-used and simple neo-Hookean hyperelastic material model. This model is known to provide a good approximation of the true material behavior when working with low strains. Its energy function for incompressible materials in 3D is described in Equation 2:

$$W_{NH}=C_{10}\left(I_{1}-3\right)$$ (2)

where *I*_1_ is the first invariant of the right Cauchy-Green deformation tensor and *C*_10_ is a material parameter representing the slope of the stress-strain curve that can be written as a function of Young’s modulus (*E*) (Equation 3).

$$C_{10}=\frac{E}{6}$$ (3)

On the contrary, we did not predict the mechanical properties of the media and adventitia layers because of the limitations of the IVUS imaging technique in accurately identifying them. As a result, the material model selected for these layers was the GOH model (Gasser et al., 2006), and their mechanical properties were selected from the literature (Latorre et al., 2022) (Table 2).

$$\begin{array}{c} \Psi =\;\mu \left(I_{1}-3\right)+\frac{k_{1}}{2k_{2}}\underset{i=4,6}{\sum }\left(\exp\left(k_{2}{\left(k\left(I_{1}-3\right)+\left(1-3k\right)\left(I_{i}-1\right)\right)}^{2}\right)-1\right) \end{array}$$ (4)

**TABLE 2:** Material parameters used in the FEA for the media and adventitia layers where the GOH strain energy was considered.

	*μ*(*kPa*)	*k*_1_ (*kPa*)	*k*_2_ (−)	*k* (−)
Media	1.4	206.16	58.55	0.29
Adventitia	8.44	547.67	568.01	0.26

All models were subject to the same boundary conditions, loading, and mesh size. In order to avoid rigid solid displacements and rotations, the model was constrained in *y* direction in a peripheral point. Due to symmetry, only half of the model was considered. A load of 18.66 *kPa* was applied, representing the high blood pressure (140 *mmHg*) of a hypertensive patient (Banegas, 2005). However, since IVUS images catch the geometrical information in an increment of 5 *mmHg* (Maurice et al., 2004), results were obtained in the last increment of 5 *mmHg* (135–140 *mmHg*). The database was generated with a full factorial approach where all the possible combinations were considered, resulting in a total of 1,782,000 cases.

From each model, several potential input variables for the ANN were obtained such as the maximum (*ϵ*_1_) and minimum (*ϵ*_2_) principal strains, the variation of the thickness of the core (*ϵ*_*core*_) and the fibrous cap (*ϵ*_*cap*_), the lumen diameter variation (*ϵ*_*theta*_) (Equation 5), and the *SR* (%). A statistical analysis was then performed to identify the most relevant variables, their significance, and multicollinearity.

$$\epsilon_{\mathrm{theta}}=\frac{\phi_{\mathrm{lum}}^{\mathrm{end}}-\phi_{\mathrm{lum}}^{\mathrm{init}}}{\phi_{\mathrm{lum}}^{\mathrm{init}}}$$ (5)

where $\phi_{\text{lum}}^{\text{end}}$ and $\phi_{\text{lum}}^{\text{init}}$ represent the lumen diameter at the end and beginning of the simulation, respectively.

### 2.2 Statistical analysis

It is widely known that Artificial Intelligence (AI) relies its predictive capacity on statistics (Gardner and Dorling, 1998). Therefore, both descriptive and inferential statistical analyses were carried out. The descriptive statistical analysis allows to detect if there is any pattern or trend followed by the candidate variables against the response variables. Complementary, the inferential statistical analysis helps in identifying those variables that most influence the response. The objective of analyzing the statistics from these two points of view is to have a better criterion to select the inputs of the ANN. For this purpose, we performed a regression analysis of each candidate variable against each response variable separately. After that, a multivariate regression analysis was executed to detect which were the most influential variables when predicting the two response variables (*E*_*core*_, *E*_*plaque*_) simultaneously. The inferential analysis was accomplished using the step-wise method with a significance of *p* < 0.05. Additionally, in order to keep the model as simple as possible, we paid special attention to the multicollinearity of the variables, using the most common method: VIF (Variable Inflation Factor). If two variables were significant but the multicollinearity factor was high, we repeated the analysis without one of the variables (Siotani et al., 1985).

### 2.3 Artificial neural network

AI is believed to be capable of giving quick responses to patient-specific clinical problems. Since our start point is the geometrical data available from the IVUS, we used a multi-layer perceptron (MLP) (Gardner and Dorling, 1998) based on supervised learning (Kotsiantis et al., 2007) to develop our Artificial Neural Network (ANN). An MLP has to extract a relation solely from the presented examples, which together are assumed to implicitly contain the necessary information for this relation (Park and Lek, 2016). The structure of an ANN consists of a collection of neurons grouped in layers, connected through weights, and activated by activation functions (Agatonovic-Kustrin and Beresford, 2000).

To develop the ANN the open-source software library for machine learning PyTorch (version 1.13.1) was used.

#### 2.3.1 Learning algorithm

The learning algorithm of the ANN starts with the division of the database into training data (80%), internal validation (10%), and testing data (10%). Next, in order to avoid one variable could predominate over the others (SR takes values of 40%–80% while strains take values of 0.5%) the data was normalized using a z-score scaler (Equation 6):

$$x_{\mathrm{scaled}}=\frac{x-\mu }{\sigma }$$ (6)

where *x* is the sample, *μ* is the mean and *σ* is the standard deviation.

Then, a forward propagation step followed by a backward propagation step is conducted (LeCun et al., 1988). In the forward propagation step, the information of each neuron (*x*_*i*_) passes to each neuron of the following layers (hidden layers) according to a weight (*w*_*i*_) previously assigned. The information passes through an activation function (*ψ*) providing the non-linearity to the network, and then, it is transferred to the following layer. Each neuron receives the sum of information from all the connected neurons activated in the previous layer. This process is summed up in Equation 7:

$$y=\psi \left(\overset{n}{\underset{i=1}{\sum }}w_{i}x_{i}+w_{\mathrm{bias}}\right)$$ (7)

where *y* is the output signal, *ψ* is the activation function and *w*_*bias*_ is the bias.

When the flow of information arrives at the last layer, it generates the outputs of the ANN, which in this study are *E*_*core*_ and *E*_*plaque*_. The weights and biases are randomly assigned in the first iteration and will be adjusted in the following iterations in order to minimize a loss function, which for the current study is the Mean Squared Error (MSE) (Equation 8).

$$MSE=\frac{1}{m}\overset{m}{\underset{i=1}{\sum }}{\left(y_{i}-\hat{y_{i}}\right)}^{2}$$ (8)

where $\hat{y_{i}}$ shows the predicted value and *y*_*i*_ shows true value where *i* = 1, 2, … , *m*.

After that, the backward propagation starts. This process relies on assigning a level of responsibility for the error to each neuron. Those neurons with a major influence in the error will have to change their weights more than those whose error’s responsibility is lower. This iterative process is the training step.

Once the training step finishes, the validation step begins. In this step, the ANN checks its capacity for predicting new values of the response variables out of the training dataset. The loss function values obtained in training and validation steps are compared in order to analyze how different they are and if the ANN suffers overfitting (Dietterich, 1995). In case they are similar and low enough, we could say the ANN has good prediction quality. In addition, in order to better quantify the error in the predictions, we computed and compared the relative error in the prediction of *E*_*core*_ and *E*_*plaque*_ for both training and testing datasets (Equation 9).

$$error=\frac{\left|\hat{y_{i}}-y_{i}\right|}{y_{i}}\cdot 100.$$ (9)

#### 2.3.2 Application to real cases

**FIGURE 3:**
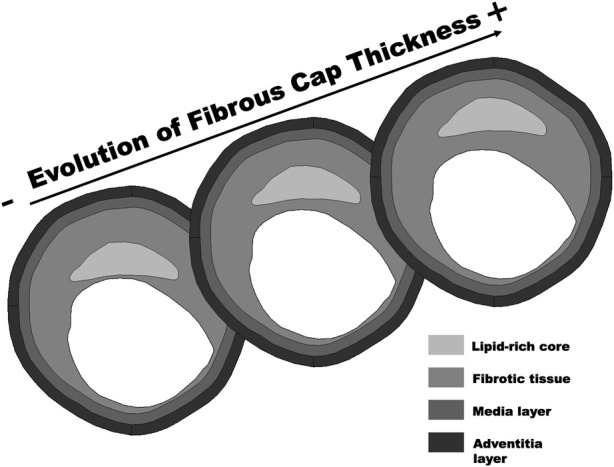
Construction of realistic database using 20 cases with varied fibrous cap thickness, based on a real geometry (65*μm*–300 *μm*).

Once the ANN was trained and tested, we applied it to 10,000 realistic geometries generated from the four real IVUS images previously published in Le Floc’h et al. (2009). The realistic *in silico* database was obtained by creating 20 different geometries varying the fibrous cap thickness from 65 *μm* to 300 *μm*, and assigning 500 different material combinations. According to this, 50 variations of *E*_*core*_ in a range of 1 *kPa*–100 *kPa* and 10 variations of *E*_*plaque*_ in a range of 390 *kPa*–1,200 *kPa* were conducted (see Figure 3).

When analyzing the predictions of the mechanical properties for the realistic geometries, the ANN predictions were not accurate (see Section 3.2). Therefore, the ANN was improved by fine-tuning the parameters using the transfer-learning technique (Tan et al., 2018; Yu et al., 2022).

Other studies have used this technique in a hybrid model based on a convolutional neural network (CNN) and a long short-term memory recurrent neural network (LSTM RNN) to classify benign and malignant breast cancer subtypes (Ikemoto et al., 2023).

#### 2.3.3 Transfer learning

Transfer learning is a machine learning technique that involves the transfer of knowledge learned in one task to improve the performance of another related task. This technique can be used to improve the prediction of the realistic dataset by leveraging the knowledge learned from a more extensive idealized dataset, improving the model’s ability to generalize to new data.

The initial layers of an MLP are responsible for identifying and extracting the fundamental and more generalized features within a dataset, whilst the later layers are supposed to learn the most specific patterns within the dataset. This is where the transfer-learning technique, known as fine-tuning, comes into play. The majority of the ANN’s weights are preserved, but the weights of the later layers are updated by re-training them with the new realistic dataset. Specifically, the last four layers of our proposed ANN were fine-tuned using the 50% of our realistic dataset, while the remaining layers were kept frozen. The other 50% of the realistic dataset was reserved for testing the ANN.

## 3 Results

### 3.1 Statistical analysis

**FIGURE 4:**
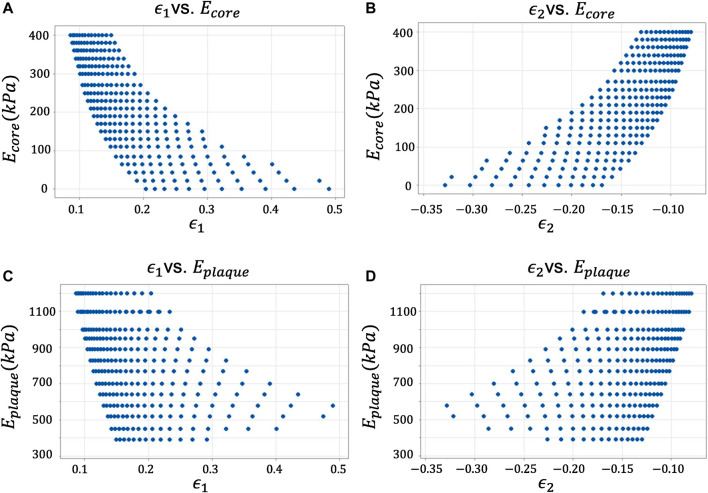
Descriptive statistical analysis of the candidate variables against the response variables. **(A)** Maximum Principal Strain *ϵ*_1_ vs. Young’s modulus of the lipid core *E*_*core*_. **(B)** Minimum Principal Strain *ϵ*2 vs. Young’s modulus of the lipid core *E*_*core*_. **(C)** Maximum Principal Strain *ϵ*1 vs. Young’s modulus of the lipid core *E*_*plaque*_. **(D)** Minimum Principal Strain *ϵ*2 vs. Young’s modulus of the lipid core *E*_*plaque*_.

Hereby, we report the results of the statistical analysis (Figure 4). In order to avoid repetitions, we show only the results of the variables *ϵ*_1_ and *ϵ*_2_ vs *E*_*core*_ and *E*_*plaque*_, since all presented a similar behavior and the conclusions drawn from them were the same. Besides, to preserve image legibility, a representative sample of the data population is reported in each plot.

As mentioned in Section 2.2, the inferential statistical analysis for the study of the most influential variables in the response was divided into: a single-output regression analysis for the prediction of *E*_*core*_ and *E*_*plaque*_ separately; and, a multivariate regression analysis for the prediction of *E*_*core*_ and *E*_*plaque*_ simultaneously. The single-output regression analysis started considering the nine candidate variables (*ϵ*_1_, *ϵ*_2_, *ϵ*_*core*_, *ϵ*_*cap*_, *ϵ*_*theta*_, $\epsilon_{\mathrm{core}}^{2}$ , $\epsilon_{\mathrm{cap}}^{2}$ , $\epsilon_{\mathrm{theta}}^{2}$ and *SR*). For *E*_*core*_ the step-wise method considered all of them but *ϵ*_2_ as significant, with $R_{\mathrm{adjusted}}^{2}=99.65\%$ , but with VIF values very high in each variable. The same was performed for *E*_*plaque*_ obtaining $R_{\mathrm{adjusted}}^{2}=99.56\%$ also with very high VIF values. After several trials, we obtained a balance between $R_{\mathrm{adjusted}}^{2}$ and VIF values. According to this, Table 3 shows the most influential variables over *E*_*core*_ and *E*_*plaque*_. Specifically, *SR* (%) and the squared variation of the thickness of the fibrous cap $\left(\epsilon_{\mathrm{cap}}^{2}\right)$ were selected for *E*_*core*_, with a $R_{\mathrm{adjusted}}^{2}$ of 90.04%, while *SR* (%) and the squared variation of the thickness of the lipid-rich core $\left(\epsilon_{\mathrm{core}}^{2}\right)$ were selected for *E*_*plaque*_, with a $R_{\mathrm{adjusted}}^{2}$ of 97.65%. Following this line, the multivariate regression analysis (Table 4) showed that the most influential variables over the two response variables simultaneously were *SR* (%), $\epsilon_{\mathrm{core}}^{2}$ and $\epsilon_{\mathrm{cap}}^{2}$ , with a $R_{\mathrm{adjusted}}^{2}$ of 92.80%.

**TABLE 3:** Summary of the best model for prediction of *E*_*core*_ and *E*_*plaque*_ separately.

*E*_*core*_			
	Coef	*p*-value	VIF
*Constant*	−161.3	0.022	-
*SR* (%)	−30.98	0.000	1.57
$\epsilon_{\mathrm{core}}^{2}$	2321.6	0.000	1.57
$R_{\mathrm{adjusted}}^{2}\left(\%\right)$	90.04		
*E*_*plaque*_			
	Coef	*p*-value	VIF
*Constant*	−4658.4	0.000	-
*SR* (%)	124.32	0.000	1.57
$\epsilon_{\mathrm{core}}^{2}$	−1,144.16	0.000	1.57
$R_{\mathrm{adjusted}}^{2}\left(\%\right)$	97.65		

**TABLE 4:** Summary of the best model for prediction of *E*_*core*_ and *E*_*plaque*_ simultaneously.

	Coef	*p*-value
*Constant*	2540	0.000
*SR* (%)	−53.62	0.000
$\epsilon_{\mathrm{core}}^{2}$	−2978	0.000
$\epsilon_{\mathrm{plaque}}^{2}$	4109	0.000
$R_{\mathrm{adjusted}}^{2}\left(\%\right)$	92.80	

According to these results, the inputs of the ANN were: $\epsilon_{\mathrm{core}}^{2}$ , $\epsilon_{\mathrm{cap}}^{2}$ , and *SR*. In addition, in order to expand the number of input parameters, we also decided to introduce $\epsilon_{\mathrm{theta}}^{2}$ .

### 3.2 Artificial neural network

**FIGURE 5:**
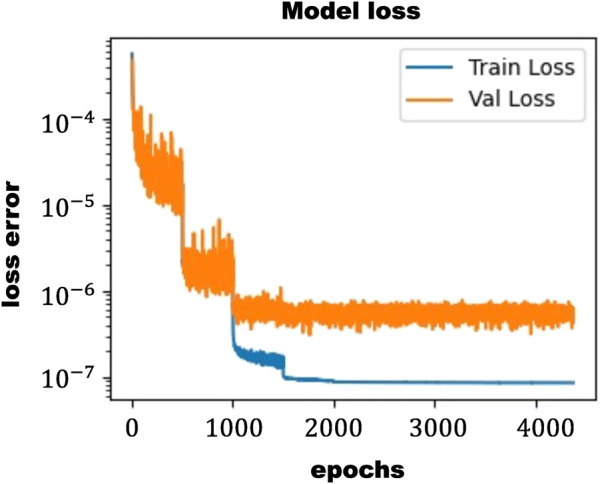
Loss function for training and validation step evaluated with the MSE.

In this study, a 14-layer ANN was employed, with rectified linear unit (ReLU) activation function applied to each hidden layer (Equation 10). The Adam optimization algorithm (Kingma and Ba, 2014) was used for training the model, and the training process was conducted over 7,000 epochs. The learning rate was dynamically adjusted along the training process, starting from 1e-3 and decreasing sequentially one order of magnitude at epochs 500, 1,000, 1,500, 2000, 3,500, and 5,000. As can be seen in Figure 5, the loss function (MSE) of this ANN achieves values under 10^–7^.

$$Relu\left(z\right)=\max\left(0,z\right)$$ (10)

To evaluate the performance of the ANN, the relative error in the prediction of *E*_*core*_ and *E*_*plaque*_ was computed in three different cases.

• First case: Original ANN tested with the idealized dataset
• Second case: Original ANN tested with the realistic dataset
• Third case: Transfer-learned ANN tested with the realistic dataset

**FIGURE 6:**
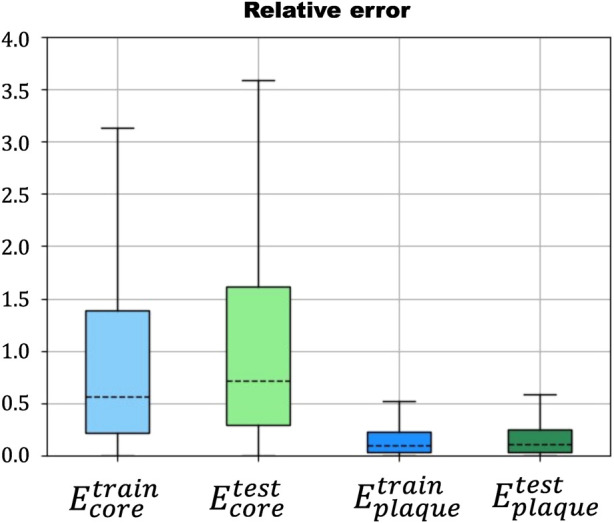
Relative error in the prediction of *E*_*core*_ (blue) and *E*_*plaque*_ (green) in the training and testing steps of the ANN.

**FIGURE 7:**
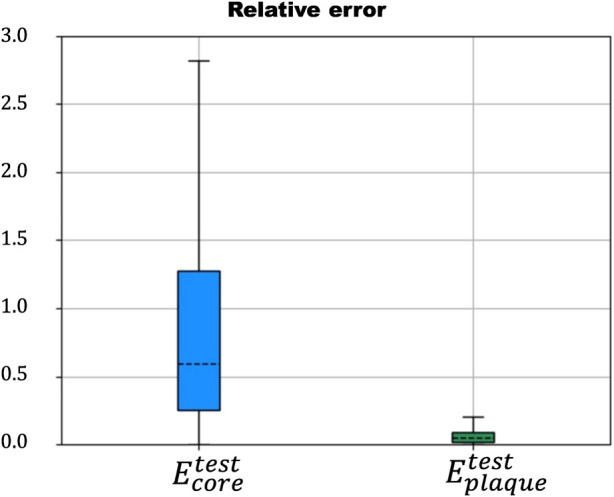
Relative error in the prediction of *E*_*core*_ (blue) and *E*_*plaque*_ (green) using the transfer-learned ANN with the realistic database.

The results of the first case showed a low relative error $\left(e_{\mathrm{train}}^{E_{\mathrm{core}}}=1.5\%,e_{\mathrm{train}}^{E_{\mathrm{plaque}}}=1.7\%,e_{\mathrm{test}}^{E_{\mathrm{core}}}=0.2\%,e_{\mathrm{test}}^{E_{\mathrm{plaque}}}=0.2\%\right)$ , indicating that the ANN was able to accurately predict the mechanical properties of the coronary artery based on the idealized geometries in the training dataset (Figure 6). The results of the second case revealed a very high relative error $\left(e_{\mathrm{test}}^{E_{\mathrm{core}}}=10^{5}\%,e_{\mathrm{test}}^{E_{\mathrm{plaque}}}=10^{3}\%\right)$ , indicating that the ANN may not perform well when applied to realistic geometries. That highlighted the need for further development of the ANN to improve its ability to predict mechanical properties on real plaques. The results of the third case revealed a relative error of less than 4% for both *E*_*core*_ and *E*_*plaque*_ when predicting realistic geometries, indicating that the ANN has been successfully fine-tuned using a small realistic dataset (Figure 7).

## 4 Discussion

In this study, we investigated the potential of artificial neural networks (ANN) for accurately estimating the mechanical properties of the atherosclerotic coronary artery. We employed a methodology that involved analyzing the statistics of the candidate variables prior to developing the ANN, which allowed us to establish a more informed criterion for selecting the inputs for the model. It is important to note that while these statistics provided useful guidance, they were not considered definitive. Given the scarcity of real-world data, our database was initially constructed using idealized geometries. To assess the ANN’s ability to generalize to more realistic scenarios, we applied it to a sample of 10,000 realistic geometries, providing a thorough evaluation of its performance.

The inferential statistical analysis was an interactive process (data not shown) where all the candidate variables were first included. In a preliminary model, almost all the candidate variables and their interactions were included as significant by the step-wise method. The model showed problems of multicollinearity, so, due to the non-linear behavior previously observed, all the linear strains were removed. Now, the step-wise method selected the *SR* (%), $\epsilon_{\mathrm{core}}^{2}$ and $\epsilon_{\mathrm{cap}}^{2}$ as significant. However, in this case, the step-wise method excluded the variable $\epsilon_{\mathrm{theta}}^{2}$ . The VIF decreased remarkably but it was still high. Therefore, in order to study which one of the multicollineal variables would better explain the response, we removed one of the variables each time. When the variable removed was $\epsilon_{\mathrm{cap}}^{2}$ the $R_{\mathrm{adjusted}}^{2}$ was 83.60% while if $\epsilon_{\mathrm{core}}^{2}$ was removed, the $R_{\mathrm{adjusted}}^{2}$ was 90.04%. In both cases, the VIF decreased to normal multicollinearity values.

The same methodology was followed for the analysis of the candidate variables over *E*_*plaque*_. The analysis with all the candidate variables and their interactions with each other and themselves considered the following variables as significant: *ϵ*_1_, *ϵ*_*theta*_, *SR* (%), $\epsilon_{\mathrm{core}}^{2}$ and $\epsilon_{\mathrm{theta}}^{2}$ . The $R_{\mathrm{adjusted}}^{2}$ was 99.56% but the VIF factor was very high, so a tuning of the model was performed. Then we considered only the squared strains and the *SR* (%). The result was a $R_{\mathrm{adjusted}}^{2}$ of 97.88% and a remarkable decrease of VIF. Since the VIF was still high, we studied the impact of removing one of the squared strains on the model, but in this case, it was not relevant because the $R_{\mathrm{adjusted}}^{2}$ was 97.65% and 97.24% on removing $\epsilon_{\mathrm{cap}}^{2}$ or $\epsilon_{\mathrm{core}}^{2}$ respectively.

Based on the statistical analysis, it was determined that the most influential input parameters for the prediction of *E*_*core*_ and *E*_*plaque*_ were $\epsilon_{\mathrm{core}}^{2}$ , $\epsilon_{\mathrm{cap}}^{2}$ , and *SR*. Additionally, to further improve the performance of the ANN, $\epsilon_{\mathrm{theta}}^{2}$ was also included as an input parameter.

The proposed ANN trained with the large and idealized database presented low relative errors in the prediction of *E*_*core*_ and *E*_*plaque*_ both during the training and the testing step. However, when applied to a realistic dataset, the ANN struggled to accurately predict the mechanical properties. To improve its performance, a fine-tuning process was applied, utilizing 50% of the realistic dataset to update the weights of the last four layers of the ANN. The result was a significant improvement in accuracy, as evidenced by a relative error lower than 3% when predicting the remaining 50% of the realistic dataset. Our results align with recent studies in the field (Torun et al., 2022), further confirming the effectiveness of using ANNs for predicting mechanical properties in the atherosclerotic coronary artery.

Other studies have also accomplished a mechanical characterization of the plaque constituents. Specifically, Akyildiz et al. (2011) studied the effects of the intima stiffness and the plaque morphology on the stress of the fibrous cap using idealized geometries based on histology images of human coronary arteries; O’Reilly et al. (2020) carried out an investigation of morphological and mechanical properties of iliofemoral and carotid atherosclerotic plaque constituents starting from *μ*CT images; Maher et al. (2009) characterized the mechanical behavior by performing tensile and compressive tests on fresh human carotid plaques removed from endarterectomy; Davis et al. (2016) studied the fracture behavior of human atherosclerotic fibrous cap using a miniature single edge notched tensile test; and, Teng et al. (2009) determined the uniaxial tensile strength of the adventitia and media of human carotid artery throughout an experimental study.

However, our novel perspective is based on the estimation of the material properties throughout a large and representative database that takes into account different lipid-rich core sizes, fibrous cap thicknesses, and stenosis ratio values.

This project is an initial step in the development of neural networks for the prediction of mechanical properties of atherosclerotic coronary arteries. There are some limitations associated. 1) The geometry used for training the ANN is an idealized geometry that only includes one lipid-rich core, which may not fully capture the complex and variable nature of real-world arterial morphology. 2) The database has been generated completely *in silico*, starting from the before mentioned idealized geometries. However, it does not exist a database of real images with mechanical properties. Therefore, we tried to overcome this limitation by applying a wide variety of mechanical properties as they can be found in the published literature. In our opinion, a possible solution for this limitation could be to develop an image-driven database. This would allow the neural network to learn directly from real cases. 3) The use of a neo-Hookean material model may be a limitation as it may not accurately capture the non-linear behavior of the plaque constituents. However, the assumption of linear material behavior is a good approximation for low Δ*P*, and in this work Δ*P* was assumed as 5 *mmHg*. 4) The study only characterized the mechanical behavior of the fibrous cap and the lipid-rich core, and did not take into account the contribution of other plaque constituents such as calcified regions.

The estimation of the mechanical properties may provide rich information to identify plaque vulnerability, such as whether an atherosclerotic coronary artery suffers from a reduced collagen synthesis, local overexpression of collagenase, or smooth muscle cell apoptosis (Libby, 2001; Akyildiz et al., 2011; Davis et al., 2016; Wang et al., 2019; O’Reilly et al., 2020). This information can also be used to evaluate the impact of different drugs in treating atherosclerosis.

As future steps, we suggest the use of 3D geometries, more complex material models for the lipid-rich core and the fibrotic tissue and if possible, a large database of images to build a neural network with different suitable architectures such as convolutional neural networks (LeCun and Bengio, 1995; Lawrence et al., 1997; Gu et al., 2018).

## 5 Conclusion

The proposed ANN model was able to accurately predict the mechanical properties of the atherosclerotic coronary artery using input parameters of $\epsilon_{\mathrm{core}}^{2}$ , $\epsilon_{\mathrm{cap}}^{2}$ , $\epsilon_{\mathrm{theta}}^{2}$ , and *SR*. The fine-tuning process applied to the ANN using a realistic dataset resulted in a significant improvement in accuracy, with a relative error lower than 3%. This work provides a novel perspective on the estimation of material properties in the atherosclerotic coronary artery through a large and representative database that takes into account different lipid-rich core sizes, fibrous cap thicknesses, and stenosis ratio values. The obtained results align with recent studies in the field, further confirming the effectiveness of using ANNs for predicting mechanical properties in atherosclerotic coronary arteries.

## Data Availability

The raw data supporting the conclusions of this article will be made available by the authors, without undue reservation.
